# A qualitative mystery shopper study that de(codes) the experiences of English and Spanish-speaking patients who call to schedule a first-time primary care appointment in the Los Angeles, Houston, and New York Metropolitan Statistical Areas

**DOI:** 10.1016/j.ssmqr.2025.100525

**Published:** 2025-01-28

**Authors:** Esmeralda Melgoza, Ahmad Ismail, Lucía Félix-Beltrán, Rosario Majano, Arturo Vargas Bustamante

**Affiliations:** a Jonathan and Karin Fielding School of Public Health, University of California, Los Angeles (UCLA), Los Angeles, CA, USA; b UCLA Latino Policy and Politics Institute, Los Angeles, CA, USA; c Research Center for Equitable Development EQUIDE, Universidad Iberoamericana Mexico City, Mexico City, Mexico

**Keywords:** Mystery shopper study, Qualitative methods, Primary care, Medicaid, Limited English proficiency, Telehealth

## Abstract

**Objective:**

To qualitatively assess the experiences that English and Spanish-speaking patients with Medicaid managed care encounter when scheduling a first-time primary care appointment, with a preference for telehealth, in the Los Angeles (LA), Houston and New York (NY) Metropolitan Statistical Areas (MSAs).

**Methods:**

A list of primary care offices was created by scraping online directories from each managed care organization (MCOs) health plan operating in the three MSAs. Primary care offices were randomly selected to participate in this qualitative mystery shopper study. Two researchers listened and transcribed real-time calls between the mystery shopper patients and the receptionists at the primary care offices. Data collection occurred between April 8, 2024, and April 26, 2024, on different days and times of the week. Both researchers completed tests to calculate the Kappa statistic, which indicated substantial inter-rater agreement. The researchers then inductively coded the transcripts using thematic analysis on Dedoose version 9.2.012.

**Results:**

Our study suggests that Spanish-speaking patients who call to schedule a first-time primary care appointment are more likely to experience call transfers, be told to call back later, and encounter more telephone hang-ups, compared to English-speaking patients. Telehealth for first-time appointments is uncommon and typically available only under special circumstances, including COVID-19, medication refills, test result reviews, and for chronically ill populations.

**Conclusion:**

This study shows disparities in access to care between English and Spanish-speaking patients at the time of scheduling a first-time appointment, highlighting an important point for future intervention.

## 1. Introduction

Medicaid is a joint federal and state program in the United States (U. S.) that provides health coverage to more than 76 million low-income adults as of February 2024. (Medicaid) Over the past several decades, the Medicaid payment system shifted from primarily fee-for-service (FFS) to managed care, although both continue to coexist. (Things to Know About Medicaid) The FFS payment system reimburses providers for each service delivered to a Medicaid beneficiary, an approach that incentivizes quantity over quality of healthcare services and results in less care coordination. (Medi-Cal Managed Care) In managed care, states contract with managed care organizations (MCOs) to provide health services to Medicaid beneficiaries in exchange for a monthly premium, or “capitation” payment, per person, an approach that incentivizes the reduction of costs, prioritizes quality over quantity of services, and increases care coordination. (Medi-Cal Managed Care) Even though MCOs receive a capitated fee, they can choose to incorporate FFS and value-based payments, such as pay-for-performance, with providers. (Things to Know About Medicaid; Share of Medicaid Population Covered; Total Medicaid MCO Enrollment) The degree to which FFS and value-based payments are incorporated by MCOs varies by state. (Things to Know About Medicaid; Share of Medicaid Population Covered; Total Medicaid MCO Enrollment)

In the U.S., there are approximately 4.9 million people with limited English proficiency (LEP) covered by Medicaid and the State Children’s Health Insurance Program (CHIP), and Spanish is the most common language among these LEP enrollees (Centers for Medicare and Medicaid Services, 2020; MACPAC, 2024). People with LEP are more likely to face challenges enrolling in and renewing their Medicaid coverage and are more likely to be disenrolled or experience gaps in their health insurance coverage, all of which contribute to poorer health outcomes, compared to their English-proficient counterparts (MACPAC, 2024). Several factors, including the lack of translated forms and the unavailability of interpreters contribute to these disparities in the healthcare system (MACPAC, 2024). Less is known, however, about the experiences that new Medicaid managed care patients with LEP encounter when they call to schedule a first-time primary care appointment, especially in the context of the rapid adoption of telehealth brought on by the corona-virus (COVID-19) pandemic.

The expansion of telehealth attempts to alleviate barriers in accessing healthcare in the U.S., with a focus on vulnerable populations, including persons with LEP. (Medicaid Enrollment and Spending Growth; Medicaid Enrollment and Unwinding Tracker; Medicaid Financing; Ding et al., 2024; Hamadi et al., 2022; Chu et al., 2021) As of January 2022, all 50 states in the U.S. covered telehealth for primary care services (Chu et al., 2021). Although use of telehealth in primary care was associated with better continuity of care and access to care (Dhaliwal et al., 2022), certain populations, including new patients and persons with LEP, continued to experience challenges in scheduling virtual appointments (Ward et al., 2022). New patients, compared to returning patients, experienced more challenges in accessing primary care telehealth appointments due to the limited ability of medical providers to conduct physical examinations and the lack of a pre-existing patient-provider relationship (Ward et al., 2022). Several studies also report that patients with LEP have lower rates of telehealth use, compared to their non-LEP counterparts (Lau et al., 2022; Rodriguez et al., 2021).

The objective of this study is to qualitatively examine the experiences that English and Spanish-speaking adult mystery shopper patients with Medicaid managed care encounter when they call to schedule a first-time primary care appointment, with a preference for telehealth, in the Los Angeles (LA), Houston, and New York (NY) Metropolitan Statistical Areas (MSAs). To our knowledge, this is the first mystery shopper study that uses qualitative methods to assess the experiences encountered by patients with LEP who are new to the Medicaid managed care system.

This study is informed by Parasuraman’s Conceptual Model of Service Quality and a mystery shopper approach, a theoretical framework and methodology that both originated in the field of marketing, with recent innovative applications in the healthcare field (Corbisiero et al., 2023; Rankin et al., 2022). Parasuraman’s Conceptual Model of Service Quality includes 10 dimensions, which are defined as follows: 1) *access*, contacting the healthcare facility is easily accessible by telephone; 2) *responsiveness*, the willingness of the receptionist to schedule timely appointments; 3) *competence*, possession of the required skills and knowledge to schedule an appointment; 4) *courtesy*, receptionists are polite, respectful, and friendly; 5) *credibility*, the receptionist is trustworthy, believable, and honest; 6) *understanding the patient’s specific needs*, makes an effort to understand the patient’s needs; 7) *communication*, maintaining patients informed in a language they can understand; 8) *tangibles*, tools or equipment used to provide the service; 9) *security*, confidentiality; and 10) *reliability*, involves consistency of performance and dependability (Parasuraman et al., 1985).

## 2. Methods

### 2.1. Overview

This study is part of a comprehensive mystery shopper study that quantitatively examined access to first-time primary care appointments, with a preference for telehealth, among Medicaid managed care beneficiaries in the LA, Houston, and NY MSAs. We partnered with a national research firm to develop scripts in English and Spanish, to train and recruit mystery shoppers, and to collect data. The sampling frame for the larger quantitative study consisted of 736 unique primary care offices across the three MSAs. Of the 736 unique primary care offices, 244 were in the Los Angeles MSA, 255 were in the Houston MSA, and 237 were in the NY MSA. The data for the comprehensive quantitative mystery shopper study were collected between April 2, 2024, and May 29, 2024. The qualitative data reported in this study were collected between April 8, 2024, and April 26, 2024, by two trained bilingual researchers who listened to the live English and Spanish calls between the mystery shopper patients and the receptionists at the primary care offices. We ended qualitative data collection when we achieved saturation of findings and no longer observed new findings from the live calls.

### 2.2. Study design and identification of medicaid managed care providers

A comprehensive list of primary care offices was created by scraping the online directories available from each of the MCOs operating in the LA, Houston, and NY MSAs. An MSA is a geographic area that has at least one urban area of 50,000 or more inhabitants. (About. Accessed August 20) The LA, Houston, and NY MSAs were selected since they have the highest share of individuals with LEP. (Overview of Health Coverage and) The LA MSA includes LA and Orange County. The Houston MSA includes Austin, Brazoria, Chambers, Fort Bend, Galveston, Harris, Liberty, Montgomery, and Waller County. The NY MSA includes counties in both the states of New York and New Jersey: Sussex, Passaic, Morris, Hunterdon, Somerset, Middlesex, Monmouth, Ocean, Bronx, New York, Queens, Richmond, and Kings. Pike County in Pennsylvania is also a part of the NY MSA but was excluded from the study because only 15.6% of the population was LEP and 7% were Spanish-speakers. Prior to the random selection of primary care offices, we stratified the lists by both MSA and language. After stratification, primary care offices were randomly selected within each strata. Sample sizes for the qualitative study were not determined a priori. We collected qualitative data until we achieved data saturation and no new findings emerged.

### 2.3. Mystery shopper call protocol

After a primary care office was randomly selected, the mystery shopper patient had access to details about their persona on a computer, including their name, gender, age, preferred language, an assigned MCO health plan, and the name, telephone number and address of a primary care provider. The persona information was preloaded and matched the mystery shopper patient’s natural and preferred presentation. MCO health plan information and office contact information were presented to mystery shopper patients randomly. They also had the script available in English-only, or both English and Spanish.

### 2.4. Qualitative data collection

The research team developed a standardized observation guide to collect the qualitative data from the live calls. The data recorded in the standardized observation guide included the unique call identification number, the name of the researcher who collected the qualitative data, the date and time of the call, the geographic location of the primary provider’s office, the caller language, and the transcripts of the call. The two bilingual researchers who collected the qualitative data listened to the live audio calls but did not participate. The collection of qualitative data occurred Monday through Friday and at different times of the day, including mornings, afternoons, and evenings. Qualitative data collection ended when data saturation was achieved, and no new findings emerged (Saunders et al., 2018).

### 2.5. Data analysis

Prior to starting the data analysis, the two researchers completed tests, one in English and one in Spanish, to calculate the Kappa statistic or inter-rater reliability score on Dedoose version 9.2.012. The Kappa statistic was K = 0.72 for the English transcripts and K = 0.84 for the Spanish transcripts, with both scores indicating substantial agreement (Gisev et al., 2013). The two researchers then inductively coded the transcripts using a thematic analysis approach, with a focus on the qualitative data produced by the receptionists. The receptionists believed they were speaking with real patients. After the first round of coding was completed, the two researchers compared their codes and subcodes, reconciled discrepancies, and created the first iteration of the codebook. A second round of coding was then conducted to compare codes and subcodes, reconcile any differences, and create the second iteration of the codebook. The second iteration was used to generate a list of themes and subthemes using a deductive data analysis approach informed by Parasuraman’s Conceptual Model of Service Quality.

### 2.6. Parasuraman’s Conceptual Model of Service Quality

We conducted an inductive thematic analysis to first identify codes and subcodes. These codes and subcodes were deductively analyzed and organized using Parasuraman’s Conceptual Model of Service Quality (see Section 3.2 and Table 3). (Parasuraman et al., 1985) The ten dimensions that comprise Parasuraman’s Conceptual Model of Service Quality were used to organize the qualitative findings of this study (see Section 3.2). The 10 dimensions are defined as follows: 1) *access*, contacting the healthcare facility is easily accessible by telephone; 2) *responsiveness*, the willingness of receptionist to schedule timely appointments; 3) *competence*, possession of the required skills and knowledge to schedule an appointment; 4) *courtesy*, receptionists are polite, respectful, and friendly; 5) *credibility*, the receptionist is trustworthy, believable, and honest; 6) *understanding the patient’s specific needs*, makes an effort to understand the patient’s needs; 7) *communication*, maintaining patients informed in a language they can understand; 8) *tangibles*, tools or equipment used to provide the service; 9) *security*, confidentiality; and 10) *reliability*, involves consistency of performance and dependability (Parasuraman et al., 1985). While 10 dimensions are available in the model, seven emerged from our data.

### 2.7. Human subjects protection

This study was approved by the institutional review board at our academic institution.

## 3. Results

### 3.1. Study sample

**Fig. 1.**
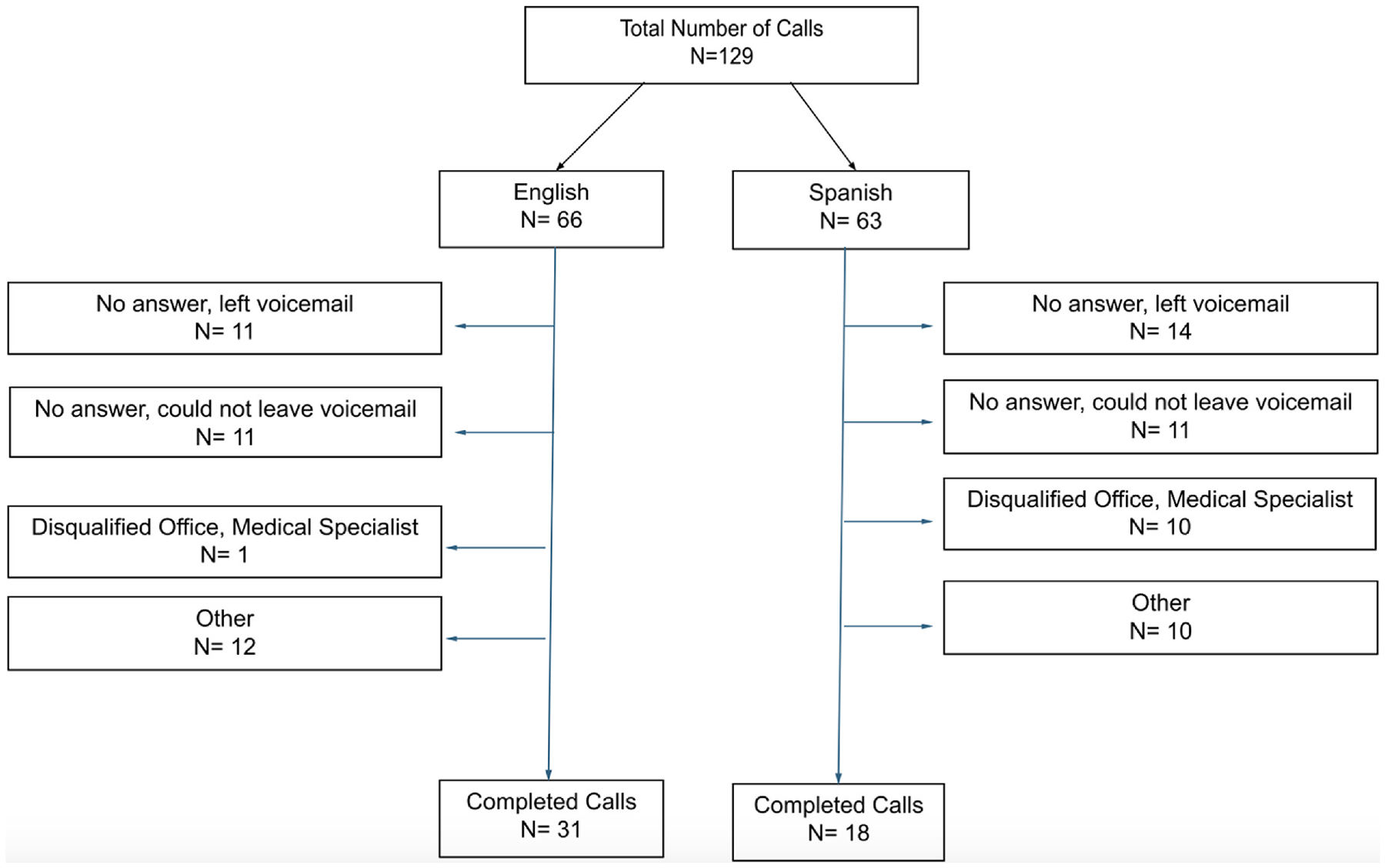
Flow chart of live calls.

The final sample for this qualitative study was 129 total calls. If a primary care office was randomly selected from the list, they were called. Calling a primary care office was not equivalent to completing a call. A completed call was defined as successfully scheduling either an in-person or telehealth primary care appointment. Of the 129 total calls, 66 and 63 calls were conducted in English or Spanish, respectively. Of the 66 calls in English, 31 (46.9%) were completed. In contrast, of the 63 calls in Spanish, 18 (28.5%) were completed. Incomplete calls, defined as calls where the mystery shoppers did not successfully schedule either an in-person or telehealth appointment were organized using the following disposition codes: 1) no answer, left voicemail; 2) no answer, could not leave a voicemail; 3) disqualified office, medical specialist, not a primary care office; 4) other reasons for not completing the call, including incorrect contact information (see Fig. 1). Table 1 shows the distribution of all calls by MSA and language. Table 2 presents the distribution of completed calls by MSA and language.

### 3.2. Themes

Based on Parasuraman’s Conceptual Model, seven themes were derived from the thematic analysis, as summarized in Table 3. Among the ten dimensions in the model, seven were observed in the data: *access, responsiveness, competence, courtesy, understanding the patient’s specific needs, communication*, and *tangibles*.

#### THEME #1: Access

##### Subtheme 1.1, 1.2, and 1.3: was a voicemail set up? What information was pre-recorded in the voicemails? Which languages were pre-recorded in the voicemails?.

Primary care offices often had pre-recorded messages available when the receptionist was not available. The messages ranged from a general message to a more specific message that instructed patients to leave their name, telephone number, and reason for calling. A few offices did not have pre-recorded messages set up, which prevented patients from leaving a voicemail. Language availability in the pre-recorded messages included English only or English and Spanish instructions. In a few cases, the voicemail systems were full, which was another barrier to leaving a voicemail.

**Pre-recorded message:** Thank you for calling XXXX Medical Group. Please leave your message and we will return your calls [Voicemail recording available in English and Spanish].

- [Transcript 1]

**Pre-recorded message:** All staff are helping other patients. Please leave a voicemail with your name, telephone number and someone will return your call [Voicemail recording available in English].

- [Transcript 3]

**Pre-recorded message:** Thank you for calling XXX Medical Group. If your call is an emergency, hang up and dial 9-1-1. We can’t get to the phone right now, but please leave your message and number, and we will call you back in 30 minutes [Recording available in English and Spanish].

- [Transcript 4]

#### THEME #2: Responsiveness

##### Subtheme 2.1: Number of times the patient is transferred because the receptionist did not speak Spanish.

The receptionist’s responsiveness was either a facilitator or a barrier to scheduling a first-time primary care appointment. Responsiveness was assessed by the number of times a patient was transferred during a call. In the LA, Houston and NY MSAs, Spanish-speaking receptionists were not uncommon. Among the primary care offices without a Spanish-speaking receptionist, however, scheduling an appointment for Spanish-speaking patients often consisted of multiple transfers. Spanish-speaking patients were also more likely to be told to call back later compared to their English-speaking counterparts.

**Patient:** Hi. Is there someone who speaks Spanish? [Call transferred].

**Receptionist:** Hi. How can I help you?

**Patient:** Yes, Is there someone who speaks Spanish?

**Receptionist:** Yes, please hold. [Call transferred again].

**Voicemail:** Please leave your name, date of birth, telephone number and a message.

**Patient:** [Proceeds to leave a voicemail]

- [Transcript 2]

**Receptionist:** Hi. How can I help you?

**Patient:** Hi. Is there someone who speaks Spanish?

**Receptionist:** Yes. you want to wait. They are busy right now.

**Patient:** Okay.

**Receptionist:** [Call transferred].

- [Transcript 2]

Subtheme 2.2: The Spanish-speaking patient was told to call back at another time

**Patient:** Hi. Is there someone who speaks Spanish?

**Receptionist:** No.

**Patient:** Okay.

**Receptionist:** You can call back after 1pm.

- [Transcript 2]

**Patient:** Hi. Is there someone who speaks Spanish?

***Receptionist:*** *No one speaks Spanish, only at night. Open at 2pm* [Receptionist responds in broken Spanish].

**Patient:** Okay, thank you. I will call later.

- [Transcript 6]

#### THEME #3: Competence

##### Subtheme 3.1: Provider name on the insurance card must match the doctor’s name.

In the LA, Houston, and NY MSAs, Medicaid managed care health plans generally include the name of the primary care provider (PCP) on members’ insurance cards. This is a common practice that helps to better coordinate care for the patient. We found that competent receptionists possessed the knowledge and skills to inform callers that the PCP’s name from their office must match the doctor’s name on the patient’s insurance card. If there was a mismatch of names, competent receptionists guided the patients on next steps. When a mismatch was found, receptionists either provided the dates for available appointments without scheduling the visits, or did not provide dates until the mismatch was resolved.

The receptionist provided dates for available appointments even when a mismatch was found

**Receptionist:** So before making an appointment, you are going to have to switch if you want to. You have to switch it [provider’s name on insurance card] for doctor X to do your test.

**Patient:** Yeah. Yeah. I can, I can, worry about that. I’ll try to get that sorted later. I’m just really trying to figure out when would be, like, when will be the next appointment right now?

**Receptionist:** Okay. So, let’s check that out. It would be, I’ll say, next Tuesday, May 7th.

- [Transcript 4]

**Receptionist:** Before you come, you have to add her as your provider. Call first and make sure she is your provider first.

**Patient:** Okay, when is the first available appointment?

**Receptionist:** Okay, let’s see. I have availability today or tomorrow [April 9 or April 10, 2024] afternoon.

- [Transcript 2]

The receptionist did not provide dates for available appointments until the mismatch was resolved

**Patient:** Ok, when would be the first available in-person appointment for a general check-up?

**Receptionist:** You would have to assign Dr. X as your PCP and then call back so we can schedule the appointment.

**Patient:** Ok, but if I could give you the number, when would be the first available appointment?

**Receptionist:** Well, that depends when the change goes into effect at your insurance company.

**Patient:** Ok, so you couldn’t tell me?

**Receptionist:** Not until I know when the change goes into effect

- [Transcript 5]

**Receptionist:** The Dr. must be assigned as the patient’s primary physician before you come to the appointment. That’s why we verify beforehand, so that none of our patients leave upset for not having verified beforehand.

**Patient:** Oh, I’ll check and call back.

**Receptionist:** Yes, there is no problem. [Translated from Spanish]

- [Transcript 6]

##### Subtheme 3.2 and 3.3: Primary care office does not accept Medicaid or the receptionist needs to check whether insurance is accepted.

Although providers were identified through online directories available through each MCO in the L.A., Houston, and N.Y. MSAs, some primary care offices did not accept Medicaid. Several conversations between patients and receptionists also suggest that some staff were unfamiliar with the differences between Medicaid managed care and specific health plans, which resulted in the use of these terms interchangeably.

**Receptionist:** What kind of insurance do you have right now?

**Patient:** HealthNet through Medicaid.

**Receptionist:** Oh, we do not accept Medi-Cal.

**Patient:** You are not taking patients with Medi-Cal?

**Receptionist:** Yes. We are not taking that insurance.

- [Transcript 4]

**Receptionist:** We have like a list of insurances, you know, in front of us, and whether we accept them or not. So, Medicaid managed care is not in front of me. So, I need to check with my manager, as to whether we accept that insurance. I can give you a call back on Tuesday.

- [Transcript 5]

#### THEME #4: Courtesy

##### Subtheme 4.1: Receptionist’s attitude towards the patient.

The receptionist’s attitude is another important facilitator or barrier to consider when patients call to schedule a first-time primary care telehealth appointment. The conversations that patients have with the receptionists are important because they set the tone for the remainder of the call and help patients navigate the complex U.S. healthcare system. There were also several instances when receptionists hung up the telephone. Telephone hang-ups were more common when the calls were made by Spanish-speaking compared to English-speaking patients.

Negative interactions

**Receptionist:** Doctor’s office.

**Patient:** Hello. Is there someone who speaks Spanish? [Receptionist hangs up the telephone]

– [Transcript 2]

**Receptionist:** Thank you for calling the doctor.

**Patient:** Hi, Is there someone who speaks Spanish?

**Receptionist:** [Hangs up the telephone].

- [Transcript 3]

**Patient:** Do you have an interpreter for the appointment?

**Receptionist:** Um, no sir.

**Patient:** Do you have telehealth appointment for general check-ups?

**Receptionist:** Yes, sir, but we don’t take that insurance. [Receptionist hangs up the telephone]

- [Transcript 5]

Positive interactions

**Receptionist:** Okay, no problem. If you have any other questions or if you are, you know, wanting to get established with one of our physicians at this office, just feel free to give us a call. We will be more than happy to get you scheduled. But you have a good day and take care.

- [Transcript 4]

**Receptionist:** I am sorry, I do not speak Spanish. would you like an interpreter?

**Patient:** Yes, please. [Brief hold to connect third party interpreter].

**Receptionist:** What is your name? [Receptionist asks the patient for their first name to be able to address her directly].

- [Transcript 4]

#### THEME #5: Understanding the Patient’s Specific needs

##### Subtheme 5.1: First primary care visit must be in person.

A challenge that patients encountered when trying to schedule a primary care telehealth appointment was that offices often required the first appointment to be in person.

**Receptionist:** Patients need to come for a face-to-face appointment before telehealth appointments.

- [Transcript 2]

**Patient:** Okay, I am assuming you do telehealth appointments for general checkups, right?

**Receptionist:** Um, we do for established patients but not for new patients.

- [Transcript 4]

**Patient:** Would I be able to get telehealth as a new patient?

**Receptionist:** No, for new patients, you have to come into the clinic.

- [Transcript 4]

**Receptionist:** If your insurance covers a virtual visit, you should be able to have a virtual check-up. Not for a new patient though.

– [Transcript 5]

**Receptionist:** The first appointment needs to be in person.

- [Transcript 6]

##### Subtheme 5.2: Telehealth available only for special circumstances.

Another challenge that patients encountered was that telehealth was reserved only for special circumstances, including the presence of COVID-19 or other infectious diseases, for medication refills, to review test results, and for chronically ill patients.

**Receptionist:** If somebody had, you know, a contagious disease or something, like, when people test positive for COVID, then we can do a virtual appointment for that. But, otherwise, most of it is in person.

- [Transcript 5]

**Receptionist:** Telehealth is only available for patients with COVID-19.

- [Transcript 4]

**Receptionist:** Medication refills can be done virtually.

- [Transcript 6]

**Receptionist:** If you want to go over results, the doctor can schedule a telehealth appointment.

- [Transcript 1]

**Receptionist:** Providers prefer telehealth appointments for very sick patients, not for general check ups.

- [Transcript 2]

##### Subtheme 5.3: Type of Provider.

Among patients who were successful in scheduling an appointment, the types of providers available included physicians, nurse practitioners, and physician assistants.

**Receptionist:** We can look, It’s your choice. We can do this with the doctor or a nurse practitioner.

- [Transcript 5]

**Receptionist:** Can I place you on a brief hold to look at the doctors available?

**Patient:** Yes, no problem.

**Receptionist:** Thank you for holding. I found a physician assistant for this upcoming Wednesday.

- [Transcript 1]

**Patient:** Do you know the kind of provider it would be with?

**Receptionist:** The provider?

**Patient:** Yeah, if it’s like a doctor or a nurse practitioner?

**Receptionist:** It would be with both.

- [Transcript 4]

#### THEME #6: Communication

Among the Spanish-speaking patients, scheduling an appointment with a provider who speaks the same language is important, although it is not always possible. Although not all primary care offices offered Spanish-speaking providers, translation services were offered in some locations. Translation services included using office staff as translators, using third-party translation services, and, in one instance, the receptionist suggested the patient bring their own translator. A few offices did not have Spanish–speaking providers nor translation services available.

**Patient:** Do you have a doctor who speaks Spanish?

**Receptionist:** This is a Latino-serving clinic, of course we speak Spanish.

- [Transcript 5]

**Patient:** Do you have a doctor who speaks Spanish?

**Receptionist:** The doctor, the nurses, and the people who work here, almost all, let’s say 99.9% of people who work here are bilingual.

- [Transcript 6]

**Patient:** Do you have doctors who speak Spanish?

**Receptionist:** No, ma’am.

- [Transcript 2]

**Patient:** Do you have a doctor who speaks Spanish?

**Receptionist:** No, but the assistants speak Spanish.

- [Transcript 3]

**Patient:** Do you have an interpreter?

**Receptionist:** It is better if you bring your own interpreter.

- [Transcript 2]

#### THEME #7: Tangibles

Tangibles refers to the tools or equipment used to provide a service, which in this context refers to the mode of telehealth delivery. Among the primary care offices that offered telehealth, the modes of delivery include telephone calls, video conferencing, or both. Some offices mentioned specific platforms, including Zoom, an application like Facetime, and calls via patient portals. This finding suggests that the modes of telehealth delivery vary across primary care offices.

**Patient:** Telehealth appointments are done with phone or video?

**Receptionist:** We are working on the video part but right now it is done through the phone.

– [Transcript 2]

**Receptionist:** Telehealth appointments are not video, but they are phone calls.

- [Transcript 5]

**Receptionist:** You can schedule telehealth appointments as either a video visit or just a phone appointment, you have an option for either.

- [Transcript 4]

**Receptionist:** We have Zoom and phone appointments available.

- [Transcript 2]

**Patient:** Like a Facetime?

**Receptionist:** It is a special application like a Facetime. The doctor will see you. We will send you a text. You open the text. You follow the instructions. The doctor will see you.

You will see him, and he will talk to you.

- [Transcript 5]

**Receptionist:** You would do it [the telehealth appointment] through your patient portal.

- [Transcript 1]

## 4. Discussion

Our study found that Spanish-speaking patients were more likely to experience call transfers, be told to call back later, and encounter more telephone hang-ups, compared to English-speaking patients who call to schedule a first-time primary care appointment. These findings suggest the importance of addressing language barriers in the primary care setting, especially with a focus on a patient’s experiences prior to their appointment with a health care provider. Most of the current research on language barriers focuses on the patient-provider encounter, without discussing the disparities that patients may experience before an appointment (Cano-Ibáñez et al., 2021; Quigley et al., 2024). Studies report that patient-provider language concordance is associated with higher patient satisfaction and comfort levels (Haskard-Zolnierek et al., 2023; Lopez Vera et al., 2023). The findings from our study suggest the importance of expanding the existing research on language barriers beyond the patient-provider encounter. For instance, setting up a voicemail with specific instructions in a language that the patient understands is important for improving access to the appointment scheduling process. Having a receptionist who speaks the same language as the patient or who is willing to contact a third-party translator also improves the appointment scheduling process. Addressing language barriers throughout the appointment scheduling process is essential to ensuring more equitable access to care. Achieving equity does not begin or stop only with the patient-provider interaction.

This mystery shopper study also suggests that patients with Medicaid managed care continue to encounter barriers when they call to schedule a first-time primary care telehealth appointment, regardless of the caller’s language. Although telehealth is approved by Medicaid for primary care visits, our findings suggest that primary care providers continue to require in-person appointments for first-time primary care visits (Chu et al., 2021). Lack of telehealth availability for first-time primary care appointments may discourage some patients without flexible work schedules from seeking care, even if eligible for Medicaid. The nature of primary care may, at least partially, explain why telehealth adoption is lower in this area of medicine compared to other specialties like psychiatry (Hsiao et al., 2023). Primary care providers typically conduct physical examinations that require in-person interactions with the patient, and are more difficult to do via telehealth. Previous studies also suggest that the adoption of telehealth is hindered by other barriers including low reimbursement rates, technical problems, a lack of investment in telecommunications infrastructure, and the need to redesign workflows to accommodate virtual appointments (Chang et al., 2021; Mold et al., 2019).

The existing literature suggests that decisions to adopt in-person, telehealth, or both options in the primary care setting are nuanced. For example, several quantitative and qualitative studies show that patients and clinicians view telehealth as appropriate for mild health conditions, chronic care management, and information gathering, but they are more likely to consider virtual appointments less suitable for severe health conditions or in situations when a thorough physical examination is required (Chu et al., 2021; Segal et al., 2022). Telehealth also has the potential to provide benefits and alleviate disparities in access to primary care for underserved populations, although researchers, practitioners, and policy makers have to intentionally place equity at the forefront (Rodriguez et al., 2021). Telehealth can alleviate transportation and opportunity cost challenges, it offers more choices to initiate care particularly for low-income newly insured individuals. Previous studies also report greater patient satisfaction for telehealth visits, compared to in-person appointments (Orrange et al., 2021; Vosburg and Robinson, 2022). Telehealth also provides a convenient entry point into the U.S. healthcare system, allowing patients to access health care in areas with limited medical resources. Future research should also explore related health care delivery issus linked to state discretionality and their specific policy guidelines, such as Current Procedural Terminology codes that influence reimbursement rates, shape health care access, as they precede practitioner discretionality.

### 4.1. Study strengths

First, the mystery shopper approach offers real-world insights into the experiences encountered by English and Spanish-speaking patients with Medicaid managed care who call to schedule a first-time primary care appointment, with a preference for telehealth. Qualitative methods provide deeper insights that complement the breadth of quantitative findings. Second, the mystery shopper methodology reduces recollection and social desirability biases that may result in under- or overreporting by respondents. Third, this study is informed by Parasuraman’s Conceptual Model of Service Quality, which provides an innovative approach to organizing the study’s findings into important themes within the U.S. healthcare system.

### 4.2. Study limitations

The first limitation is that our study findings are based on qualitative data collected in the LA, Houston, and NY MSAs, which may not be representative of other geographic areas in the U.S. This study, however, covers a larger geographic area compared to previous mystery shopper studies (Grande et al., 2018; Primary Care Access to New). The second limitation is that live calls were not audio or video recorded, due to state laws which prohibit recording without the consent of all parties involved. While this hindered our ability to cross-check our data, the researchers collecting the data conducted a review immediately after the calls to ensure high data quality. (Recording Phone Calls and Conversations)

## 5. Implications for health services research, practice, and policy

To address language barriers in scheduling appointments, we recommend providing guidance to primary care offices regarding the content of their pre-recorded messages. For example, in this study, pre-recorded messages with specific instructions were more effective than general messages. The pre-recorded messages prompted patients to provide their name, telephone number and a brief explanation of their concern. It is also important to make the pre-recorded messages available in multiple languages that best reflect the communities served by the primary care offices. If there are language barriers between the patient and the provider or their staff, third-party interpreters should be readily available.

Findings from this study suggest that there are several barriers to scheduling a first-time primary care telehealth appointment for adult patients with Medicaid managed care in the LA, Houston, and NY MSAs. The first major barrier is that telehealth is often not the preferred mode of healthcare delivery for providers, particularly in the context of first-time primary care appointments for new patients. We propose several changes that may increase confidence in the use of telehealth for first-time primary care appointments. First, primary care offices may choose to incorporate a brief electronic or telephone intake prior to the initial telehealth appointment to collect patient information and streamline the process. Second, healthcare providers can leverage remote patient monitoring technologies, telephone applications, and smartwatches, to obtain patient information, including weight, height, and vitals, eliminating or reducing the need for an initial in-person visit. Third, supporting and training clinicians on best practices to conduct virtual medical examinations may increase the confidence of primary care providers in conducting telehealth appointments (Frehn et al.).

## 6. Conclusion

Our study suggests that Spanish-speaking patients are more likely to experience call transfers, be told to call back later, and encounter more telephone hang-ups, compared to English-speaking patients who call to schedule a first-time primary care appointment. These findings highlight the importance of addressing language barriers prior to the patient-provider appointment, which hinder a person’s ability to access healthcare in the U.S. Overall, telehealth for first-time appointments is also rarely available, with offices often requiring an in-person visit first. First-time telehealth appointments are commonly offered only for special circumstances, including COVID-19, medication refills, test result reviews, and for chronically ill populations.

## Figures and Tables

**Table 1:** Distribution of all calls based on language and MSA.

	English	Spanish	Total
Los Angeles	34	26	60
Houston	15	27	42
New York	17	10	27
Total	66	63	129

**Table 2:** Distribution of completed calls based on language and MSA.

	English	Spanish	Total
Los Angeles	17	6	23
Houston	5	8	13
New York	9	4	13
Total	31	18	49

**Table 3:** Conceptual Model, themes, and subthemes.

Themes, according to Dimensions from Parasuraman’s Conceptual Model of Service Quality	Definition of Dimensions/Themes	Subthemes
1. Access	Contacting the healthcare facility is easily accessible	1.1 Was a voicemail set up? 1.2 What information was pre-recorded in the voicemails? 1.3. What languages were pre-recorded in the voicemails?
2. Responsiveness	Willingness of receptionist to schedule timely appointments	2.1 The number of times the call is transferred before scheduling an appointment
3. Competence	Possession of the required skills and knowledge to schedule an appointment	3.1 Provider name in the insurance card must match the doctor’s name in the office. 3.2. Primary care office does not accept Medicaid. 3.3 Receptionist needs to check whether insurance is accepted.
4. Courtesy	Receptionists are polite, respectful, and friendly.	4.1 The receptionist’s attitude towards the patient
5. Understanding the Patient’s Specific Needs	Makes an effort to understand the patients’ needs and specific requirements.	5.1 First time primary care appointment must be in person 5.2 Telehealth for special circumstances 5.3 Type of provider
6. Communication	Maintaining patients informed in a language they can understand and listen to them	6.1 Was there a Spanish-speaking doctor available? 6.2 Are there translation services available for Spanish-speaking patients?
7. Tangibles	Tools or equipment used to provide the service	7.1 Format of telehealth appointment
8. Credibility	The receptionist is trustworthy, believable, and honest.	These dimensions were not present in the qualitative data analyzed for this study.
9. Security	Confidentiality	These dimensions were not present in the qualitative data analyzed for this study.
10. Reliability	Involves consistency of performance and dependability	These dimensions were not present in the qualitative data analyzed for this study.
